# Cost-effectiveness of atrial fibrillation screening programmes across European nations

**DOI:** 10.1093/ehjqcco/qcaf099

**Published:** 2025-08-25

**Authors:** Lars Bernfort, Johan Lyth, Kajsa Appelberg, Giuseppe Boriani, Claire M Buckley, Søren Zöga Diederichsen, Michaela Eklund, Daniel Engler, Ben Freedman, Tatjana S Potpara, Renate B Schnabel, Jesper Hastrup Svendsen, Lars-Åke Levin

**Affiliations:** Department of Health, Medicine and Caring Sciences, Linköping University, Campus US, Linköping 581 83, Sweden; Department of Health, Medicine and Caring Sciences, Linköping University, Campus US, Linköping 581 83, Sweden; Department of Health, Medicine and Caring Sciences, Linköping University, Campus US, Linköping 581 83, Sweden; Cardiology Division, Department of Biomedical, Metabolic and Neural Sciences, University of Modena and Reggio Emilia, Policlinico di Modena, 411 24 Modena, Italy; School of Public Health, University College Cork, T12K8AF Cork, Ireland; Department of Cardiology, The Heart Centre, Copenhagen University Hospital—Rigshospitalet, 2200 Copenhagen, Denmark; Department of Health, Medicine and Caring Sciences, Linköping University, Campus US, Linköping 581 83, Sweden; Department of General and Interventional Cardiology, The University Medical Center Hamburg-Eppendorf, 202 51 Hamburg, Germany; Heart Research Institute, Charles Perkins Centre and Faculty of Medicine and Health, The University of Sydney, NSW 2042 Sydney, Australia; School of Medicine, University of Belgrade, 11000 Belgrade, Serbia; Department for Intensive Arrhythmia Care, Cardiology Clinic, Clinical Center of Serbia, 11000 Belgrade, Serbia; Department of General and Interventional Cardiology, The University Medical Center Hamburg-Eppendorf, 202 51 Hamburg, Germany; Department of Cardiology, The Heart Centre, Copenhagen University Hospital—Rigshospitalet, 2200 Copenhagen, Denmark; Department of Clinical Medicine, Faculty of Health and Medical Sciences, University of Copenhagen, DK-1165 Copenhagen, Denmark; Department of Health, Medicine and Caring Sciences, Linköping University, Campus US, Linköping 581 83, Sweden

**Keywords:** Anticoagulants, Atrial fibrillation, Cost-effectiveness, Health care, Screening, Stroke

## Abstract

**Aims:**

Due to the ageing population in Europe, a significant increase in the incidence of atrial fibrillation (AF) is anticipated. This is predicted to have a detrimental impact on public health costs, particularly among the elderly, because of an increased number of stroke cases. Early detection of AF is crucial for initiating treatment with oral anticoagulants (OACs) to reduce the risk of stroke. This study aims to assess the cost-effectiveness of implementing AF screening programmes in eight European countries: Denmark, Germany, Ireland, Italy, the Netherlands, Serbia, Spain, and Sweden.

**Methods and results:**

The analysis concerned invitation to AF population screening for 75-year-olds. A Markov cohort model was used, considering the prevalence of AF, screening yield, the use of different OACs, estimated clinical events, mortality, quality of life and costs. The model used country-specific parameters to produce specific cost-effectiveness estimates. Probabilistic sensitivity analyses were conducted to assess the impact of parametric uncertainties on the results. Inviting 75-year-olds to AF screening proved to be cost-effective across all eight countries analysed. In all countries, the strategy was dominant, meaning that quality-adjusted life-years were gained at lower costs. The time to financial break-even ranged from 6 to 14 years.

**Conclusion:**

This study indicates that population-based AF screening of 75-year-olds is a cost-effective strategy across eight European countries, meaning that adoption of such a strategy has the potential to make healthcare systems in these countries more efficient. The heterogeneity among European countries suggests that AF screening programmes may need to be tailored to the specific healthcare systems and conditions of each nation.

## Graphical Abstract

**Graphical Abstract:**
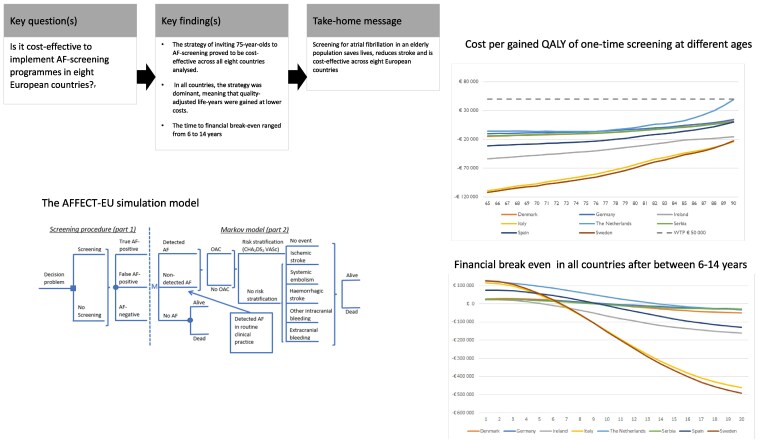

Key learning points What is already known:

- Based on previous studies, there are indications that AF screening is cost-effective in the countries studied.

What this study adds:

- This is the first pan-European analysis, demonstrating the cost-effectiveness of population screening for AF in eight different countries.
- Although AF-screening is cost saving in all analysed countries, differences between countries causes differences in time to financial break-even.
- Health benefits differ between countries due to differences in survival, quality of life, and anticoagulation treatment.

## Introduction

Atrial fibrillation (AF) is associated with increased mortality and morbidity due to an increased risk of ischaemic stroke,^1^ but also giving rise to other adverse outcomes, such as hospitalization, cognitive decline, heart failure, resulting in important disability,^2^ as well as reduced survival. The incidence of AF increases with age and exceeds 3% in Europe in the adult population.^3-6^ Due to the aging European population the prevalence of AF in Europe is expected to double by 2060.^7^ Oral anticoagulation (OAC) therapy, either direct oral anticoagulants (DOAC) or vitamin K antagonists (VKA), can effectively reduce the risk of stroke in AF patients.^8^ The European Society of Cardiology guidelines recommend early detection of AF to prevent complications and optimize treatment, including the use of effective OAC.^9^ The guidelines also recommend heart rhythm assessment during healthcare contact for people over 65 years and systematic screening for people over 75 years and/or with a high risk of stroke.

Previously published economic evaluations have demonstrated AF screening to be cost-effective regardless of strategy and method.^10-22^ The cost-effectiveness results in previous studies were driven by the efficacy of the programme in terms of discovering new AF patients, and compliance to anticoagulation treatment which would impact cardio-embolic stroke from AF.

To optimize screening for AF it is important to implement risk-based, cost-optimized AF screening strategies. Optimal strategy may differ between countries, so it is not feasible to evaluate the cost-effectiveness of AF screening in a single pan-European calculation. Reasons why cost-effectiveness differs between countries are, for example variations in health care costs and resource use, differences in treatment traditions, epidemiology context such as factors that influence incidence and prevalence, demographic context, life expectancy. Heterogeneity renders it problematic to apply and compare cost-effectiveness findings uniformly across countries.^23,24^

Wahler *et al*.^25^ analysed the cost-effectiveness of a photoplethysmography procedure for AF screening in six European countries. They found that in all six countries the number of strokes decreased, and quality-adjusted life-years increased. The higher the healthcare costs in a country, the more favourable was the cost-effectiveness.

As a part of the European collaboration project AFFECT-EU,^26^ this study aims to assess the cost-effectiveness, from a healthcare perspective, of implementing population-based AF screening in eight countries across Europe: Denmark, Germany, Ireland, Italy, the Netherlands, Serbia, Spain and Sweden and to compare the results.

## Methods

The base-case analysis concerns population screening of 75-year-olds, as this is the recommended age for population screening according to guidelines from the European Society of Cardiology.^9^ In all countries, except for Sweden, single time recordings are used. In Denmark, Germany, Ireland, and Serbia a 12-lead electrocardiogram (ECG) interpreted by cardiologist is used as screening device. In Italy measurement by use of smartphone (interpreted by cardiologist) is applied. In the Netherlands and Spain, the results from handheld ECG are interpreted using an algorithm. In Sweden, measurement twice daily for 2 weeks using handheld ECG interpreted by cardiologist is applied. Extended analyses include people 65 years and older. In the base-case analysis, the same stroke risk is assumed for all AF patients. Due to uncertainty regarding the risk of stroke with screen-detected AF compared with clinically diagnosed AF, we conducted a sensitivity analysis in which the stroke risk with screen-detected AF was assumed to be 25–50% lower than with clinically diagnosed AF. Population screening is assumed in analyses of all countries, to render results comparable. Since currently, no systematic population screening is implemented in any of the countries, the screening strategy is compared with no screening. The nature of AF, affecting mortality risk and with a lifelong impact on individuals, requires the use of a lifetime horizon for the analysis. Based on published data, a state-transition Markov model with 3-month cycles was used to simulate outcomes over a lifetime horizon. The primary outcome of the analysis is cost-effectiveness in terms of cost per quality-adjusted life-year (QALY). Other significant outcomes reported were stroke incidence and mortality.

### The AFFECT-EU Markov model

**Figure 1:**
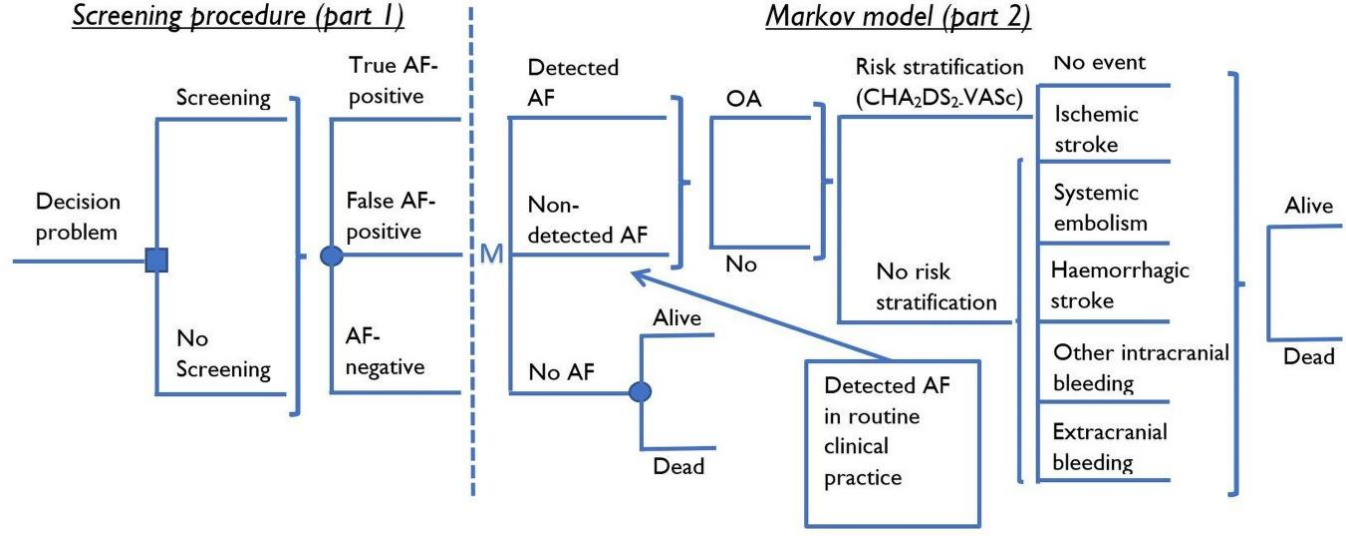
The AFFECT-EU model, description of health states and clinical events for screen-detected atrial fibrillation patients.

The decision analytic model used for the cost-effectiveness analyses consists of two parts, a decision tree describing the initial screening procedure and a Markov model tracking long-term health related consequences and costs. The structure of the model is shown in *Figure 1*.

The first part of the model determines the number of screening-detected persons, by use of age-specific prevalence of AF and sensitivity of the applied screening device. The Markov model describes anticoagulation treatment and the risk of stroke. In the Markov model, patients begin in either of the states *Detected AF*, *Non-detected AF* or *No AF*. In case of AF, patients either receive *OAC treatment* or not (*No OAC*). Depending on whether AF patients receive OAC or not, they experience risks of clinical events; *ischaemic-* or *haemorrhagic stroke*, *systemic embolism*, *other intracranial bleeding*, *or extracranial bleeding*.

### Model parameters

Parameters applied in the model analysis are summarized in *Table 1*.

**Table 1:** Parameters applied in the AFFECT-EU model

Parameter	Mean	Comment	Source
**Baseline characteristics**			
Age (years)	75		
% Female	52		
% Invited screened	51		^26^
AF prevalence		Age and sex dependent	See *Table 2*
Screening yield		Age and sex dependent	See *Table 2*
CHA_2_DS_2_-VASc score		Age and sex dependent	See *Table 3*
Treatment cessation rates		Country specific and time dependent	See Supplementary material online, Table *S1*
**Probabilities (risks)** ^a^			
Ischaemic stroke		Depends on age, sex, CHA_2_DS_2_-VASc, OAC treatment	See *Table 4*
Haemorrhagic stroke		Depends on OAC treatment	See *Table 5*
Systemic embolism		Depends on OAC treatment	See *Table 5*
Other intracranial bleeding		Depends on OAC treatment	See *Table 5*
Extracranial bleeding		Depends on OAC treatment	See *Table 5*
OAC treatment (DOAC/VKA/No)		Country-specific distribution	See *Table 6*
Stroke-related mortality		Dependent on time after stroke event, age adjusted. IS and HS resp.	See Supplementary material online, table *S2*
Standard mortality		Country specific, age and sex adjusted	See Supplementary material online, Table *S3*
Costs		Country specific	See Supplementary material online, Table *S4*
Utilities (quality of life weights)		Country specific, age adjusted population weights	See Supplementary material online, Table *S5*
Utility decrements		After stroke (ischemic stroke and haemorrhagic stroke). Depends on time after event	See *Table 7*

^a^Risks for clinical events are per 3 months.

The prevalence of known AF by age and sex is based on the average prevalence of published studies from seven countries.^3-6,27-29^ Smoothing with linear interpolation is used to obtain 1-year prevalence out of the original prevalence (5- or 10-year intervals). Screening yield is the expected proportion of previously unknown AF found by screening. Screening yield by sex and age is taken from a large meta-analysis including studies using single timepoint ECG.^30^ See *Table 2* for details.

**Table 2:** Prevalence of known atrial fibrillation and screening yield, for men and women in different ages

Age	Prevalence known AF—Men (%)	Prevalence known AF—Women (%)	Screening Yield—Men (%)	Screening Yield—Women (%)
65	5.0	2.7	1.1	0.7
66	5.6	3.1	1.2	0.7
67	6.2	3.4	1.3	0.7
68	6.9	3.9	1.4	0.7
69	7.6	4.4	1.5	0.8
70	8.3	4.9	1.6	0.9
71	9.0	5.3	1.7	0.9
72	9.7	5.8	1.8	1.0
73	10.6	6.4	1.9	1.1
74	11.4	7.0	2.0	1.2
75	12.3	7.7	2.1	1.3
76	13.2	8.4	2.2	1.5
77	14.0	9.1	2.3	1.6
78	14.7	10.0	2.4	1.6
79	15.4	11.0	2.5	1.7
80	16.1	11.8	2.5	1.8
81	16.9	12.8	2.6	1.8
82	17.5	13.6	2.7	1.9
83	18.0	14.3	3.0	2.2
84	18.5	15.1	3.2	2.5
85+	19.0	15.7	3.5	2.8

Risk stratification for ischaemic stroke was based on CHA_2_DS_2_-VASc, no risk stratification was made for other events. The age- and sex-specific distribution of CHA_2_DS_2_-VASc scores was estimated from the LOOP study.^31^ See *Table 3* for details.

**Table 3:** Age- and sex-specific CHA_2_DS_2_-VASc scores, for men and women in different age intervals

Age/CHA_2_DS_2_-VASc	2	3	4	≥5
**Men**				
65–69	36%	33%	19%	12%
70–74	36%	33%	19%	12%
75–79	0%	36%	39%	25%
80–84	0%	36%	36%	29%
85–110	0%	57%	29%	14%
**Women**				
65–69	0%	47%	23%	30%
70–74	0%	47%	23%	30%
75–79	0%	0%	66%	34%
80–84	0%	0%	50%	50%
85–110	0%	0%	57%	43%

Risks of ischaemic stroke^32-38^ are presented in *Table 4*, and risks of other events^32-35,37-41^ are described in *Table 5*.

**Table 4:** 3-Months risk of ischaemic stroke shown by CHA_2_DS_2_-VASc score

	CHA_2_DS_2_-VASc (%)					
	0	1	2	3	4	≥5
DOAC	0.01	0.04	0.15	0.22	0.33	0.63
VKA (Warfarin)	0.01	0.04	0.16	0.24	0.36	0.69
No treatment	0.04	0.11	0.45	0.68	1.01	1.92

**Table 5:** 3-Months risk of haemorrhagic stroke, systemic embolism, other intracranial bleeding, and extracranial bleeding

	Haemorrhagic stroke (%)	Systemic embolism (%)	Other intracranial bleeding (%)	Extracranial bleeding (%)
DOAC	0.06	0.02	0.05	0.62
VKA (Warfarin)	0.12	0.02	0.08	0.69
No treatment	0.04	0.11	0.02	0.39

Standard mortality data was adapted from The Human Mortality Database^42^ and increased mortality risk after stroke comes from a Swedish study.^43^ Data on OAC treatment effects and safety comes from clinical trials^44,45^ and meta-analyses.^27,28^ Proportion of patients treated with different OACs (DOAC or VKA) was assumed based on a mix of published studies and personal communication with country-specific experts, see *Table 6*.

**Table 6:** Utility decrements applied in the AFFECT-EU model analyses

State/event	Utility weight decrement
**Utility loss due to ischaemic stroke**	
0–6 months	0.19
7–12 months	0.15
13–24 months	0.15
25–60 months	0.19
60+ months	0.19
**Utility loss due to haemorrhagic stroke**	
0–6 months	0.27
7–12 months	0.20
13–24 months	0.18
25–60 months	0.040
60+ months	0.070

### Resource use and unit costs

Resource use and unit costs, i.e. cost data, were based on a mixture of published studies,^46-53^ official price lists and expert assumptions. Cost parameters in the model were adjusted for country-specific healthcare inflation to 2023 by Harmonised Indices of Consumer Prices by Classification of Individual Consumption by Purpose (Coicop)-divisions.^54,55^ In cases when country-specific costs were not available, costs parameters were adjusted with healthcare purchasing power parities^56^ to translate from a country with available cost to the country of interest. The Danish and Swedish costs were recalculated to Euros by using the average August 2023 country-specific exchange rates.^57^ Due to the age structure of the patient population a healthcare perspective was applied, meaning that production costs were excluded from the analysis. For a summary of the costs applied, see Supplementary material online, Table  *S4*.

### Utility weights

Country-specific age adjusted (65 to 85+) population utility weights were used in the analysis.^58,59^ Utility decrements due to post-stroke states were obtained from published studies^60,61^ and are presented in *Table 7*.

**Table 7:** Summary of country-specific results, per 1000 persons invited to atrial fibrillation screening compared with no screening

	Strokes avoided	Life-years gained	QALYs gained	Additional costs (€)	ICER (€/QALY)
Denmark	3.4	3.3	5.5	−56 065	Dominant
Germany	2.8	2.4	4.1	−29 667	Dominant
Ireland	2.8	2.5	4.1	−167 934	Dominant
Italy	3.3	3.5	5.9	−494 753	Dominant
The Netherlands	2.8	3.3	5.7	−37 326	Dominant
Serbia	2.3	1.6	3.1	−30 666	Dominant
Spain	3.4	3.7	6.1	−147 084	Dominant
Sweden	3.5	3.8	6.0	−531 474	Dominant

The cycle length used in the Markov model analysis was 3 months, without half-cycle correction due to the short cycle-length. The analytic perspective applied was that of the health care sector and the discount rates applied for costs and QALYs varied between countries, see Supplementary material online, Table  *S6*.

### Cost-effectiveness

The effects of AF screening on costs and QALYs were used to calculate cost-effectiveness in terms of incremental cost-effectiveness ratios (ICERs). An ICER is calculated as incremental costs divided by incremental QALYs for AF screening compared with no AF screening.

## Results

In all eight countries analysed of the strategy of inviting all 75-year-olds to AF screening is cost-effective compared with no screening. In all analysed countries AF screening is even a dominant strategy, i.e. it both gains health (QALYs) and saves costs.^62^ In *Table 7* the results are summarized per 1000 persons invited to screening, with respect to strokes avoided, life-years gained, QALYs gained, additional costs with screening, and the country-specific ICERs. Scatter plots describing results of probabilistic cost-effectiveness analyses for each country are presented in the supplement (see Supplementary material online, *Figures S1*–*S8*).

**Figure 2:**
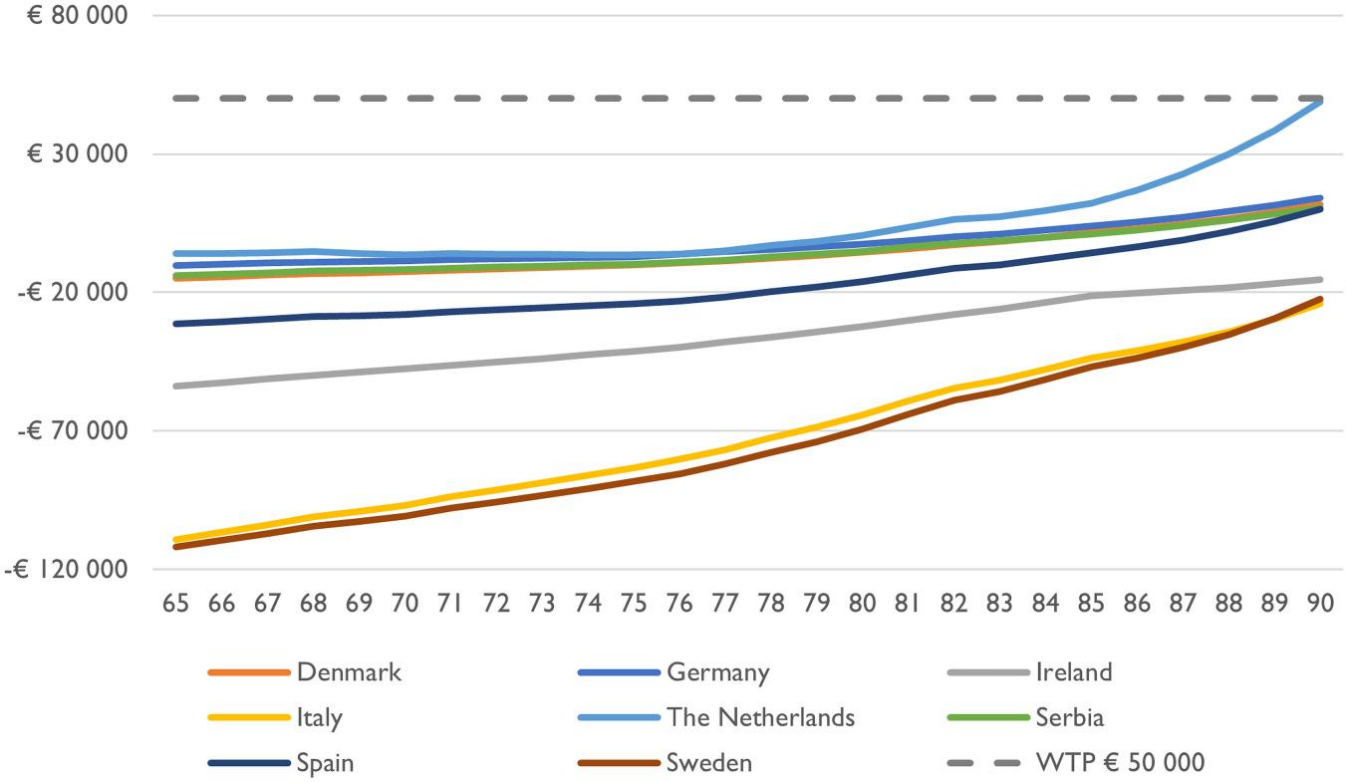
Incremental cost (*y*-axis) per quality-adjusted life year of one-time screening at different ages (*x*-axis) in the eight countries.

Results differ between countries due to variations in the underlying country-specific parameters. Deterministic sensitivity analyses showed robust cost-effectiveness results, and no single parameter had a decisive effect on the result when varied. Probabilistic sensitivity analyses showed that AF screening is probably cost-effective. Applying a threshold of €17 000 per QALY, AF screening is with 99% certainty cost-effective in all the eight countries. For details, see Supplementary material online, *Figures S1*–*S8*. The country-specific ICERs when applying one-time screening at different ages are summarized in *Figure 2*.

**Figure 3:**
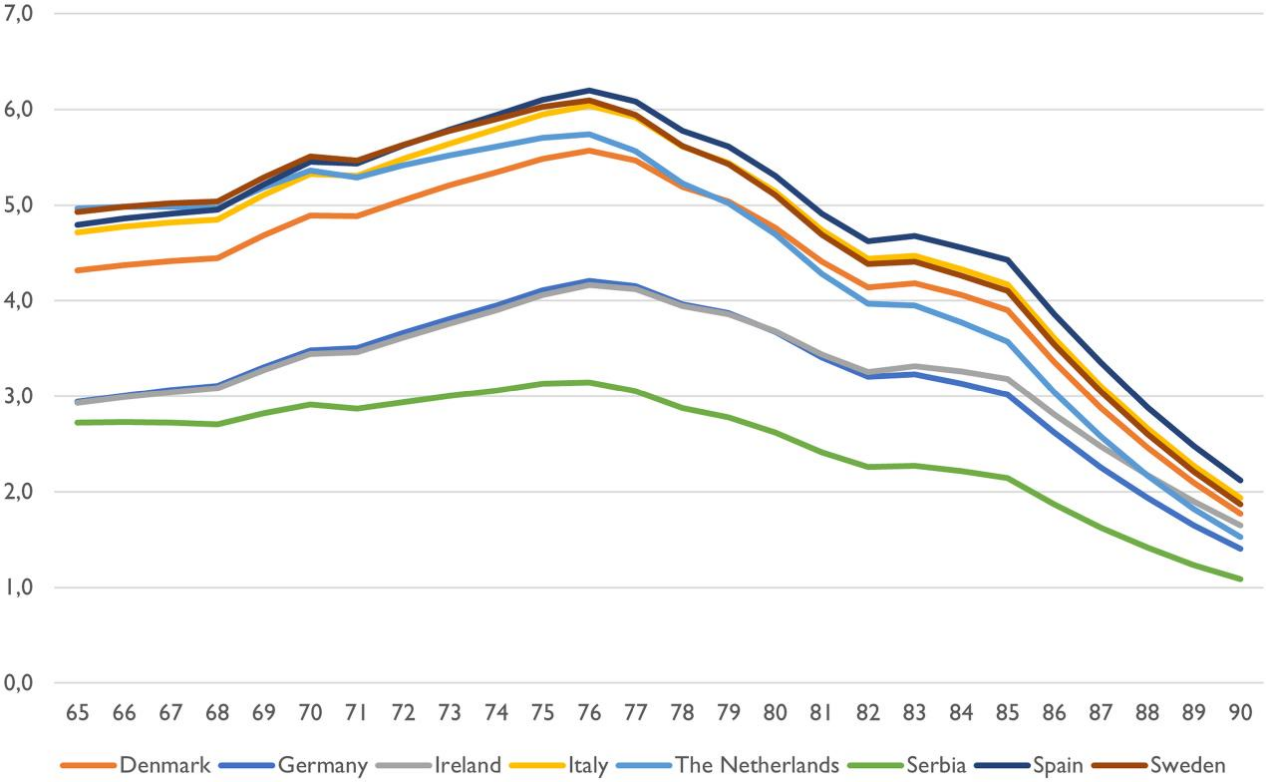
Country-specific incremental health gains (quality-adjusted life years, on the *y*-axis) of one-time screening at different ages (*x*-axis), per 1000 invited individuals.

To determine the optimal age for one-time screening, it is essential to analyse at which age screening produces the greatest health gains (QALYs) compared with no screening. In *Figure 3*, the optimal screening ages that maximize health gains for the eight countries are presented.

**Figure 4:**
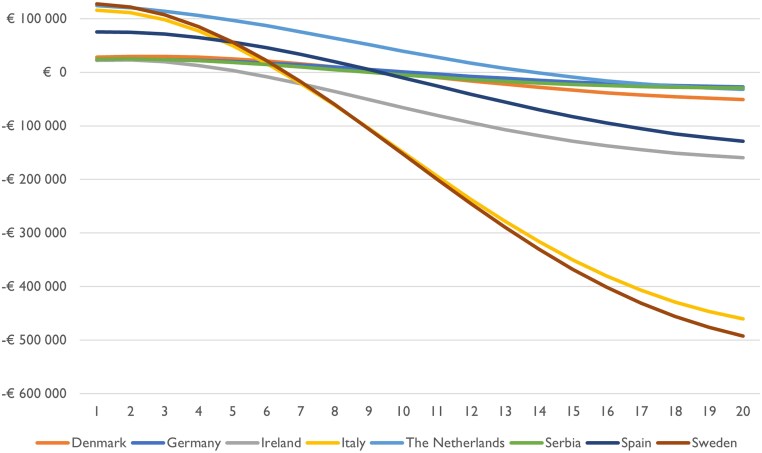
Country-specific aggregated incremental costs (*y*-axis) of atrial fibrillation screening in different ages (*x*-axis), per 1000 invited individuals.

Even though the level of health gain differs between countries, they are uniform in that the optimal screening ages are gathered around 76 years. The timeline for achieving cost savings from AF screening varies across countries, as illustrated in *Figure 4*. This figure illustrates the country-specific aggregated incremental costs of AF screening at different ages, per 1000 invited individuals.

In all countries, the break-even point is attained within a timeframe of 6–14 years, with Ireland reaching break-even in 6 years and the Netherlands in 14 years. This means that on an aggregated level costs are saved when using a time-horizon of 6–14 years.

A sensitivity analysis regarding stroke risk in screen-detected AF showed that in all eight countries except for Serbia, AF screening is a cost-effective strategy when reducing the stroke risk by 50% compared with the risk in clinically diagnosed AF. See Supplementary material online, *Table S8* for the full results of the sensitivity analysis.

## Discussion

The model-based cost-effectiveness analyses indicate that invitation of 75-year-olds to population-based AF screening is cost-effective in all the eight analysed countries. In all countries, the point estimates of incremental costs and QALYs of screening invitation (compared with no invitation) reveal that the screening strategy is not only cost saving but also enhances health outcomes.

The analyses indicate that AF screening is almost certainly cost-effective across the eight countries. Applying a threshold of €17 000 per QALY, AF screening is with 99% certainty cost-effective in all the eight countries. Notably, the optimal age for screening, where the health benefits are maximized, is identified as 76 years in all analysed countries. These observations extend and help generalize data from the STROKESTOP study, which analysed population-based AF screening of 75/76-year-olds.^18^ Main drivers influencing the cost-effectiveness results across all countries were predominantly costs related to stroke care, followed by costs associated with screening devices and anticoagulant treatment.

Previously published cost-effectiveness studies have almost exclusively concluded that AF screening is cost-effective compared with no screening. Several previous studies have, just like this study, concluded that AF screening is cost saving.^17-20^

Sensitivity analyses indicated that the results were robust, with no single parameter significantly altering the outcome when varied. Additionally, probabilistic sensitivity analyses demonstrated that AF screening is almost certainly a dominant strategy in Sweden, Ireland, and Italy. In Denmark, Germany, the Netherlands, Serbia, and Spain AF screening is likely dominant.

The screening strategy analysed in this study was, for all eight countries, (invitation to) population-based screening of 75-year-olds. This specific strategy might not align with the preferred screening strategy in each country. Therefore, the analyses may need to be adjusted to better fit the actual health policies of the respective countries. In this study, screening by single-time 12-lead ECG interpreted by cardiologist was analysed as we believe this to be the most feasible method for broad implementation. In comparison with for instance implantable loop recorder or a multiday Holter, this strategy is less costly but can be expected to pick up less AF. For the reason of comparability, the same strategy was analysed across countries. The method actually used in Ireland is pulse palpation as initial screening mode and then a 12-lead ECG is undertaken in some instances. This means that the costs, but also the health benefits concerning the Irish case might be slightly over-rated. For all countries, we used a large meta-analysis for estimating age- and sex-specific screening yield based on single timepoint measurement. This is likely to provide a good basis for most countries that use single timepoint measurement. For Sweden, using repeated timepoint measurement, the screening yield is probably somewhat underestimated.

The varied healthcare systems, costs, demographics, and public health policies across European countries make it challenging to calculate a meaningful common pan-European ICER for AF screening. However, within this cross-European analysis we could interpret that specific regional health care settings and practice may have an impact of the ICER and obviously the implementation of an AF screening approach. It is therefore imperative to evaluate each country on an individual basis, as variations in treatment protocols and epidemiology between countries may significantly influence both the estimated health benefits and costs associated with screening. The health benefits, quantified in quality-adjusted life years (QALYs) gained, are adversely affected in countries with a lower life expectancy,^24^ and cost savings are larger in countries with high healthcare costs.^25^ Similarly, in countries where OAC treatment in AF still includes a relatively high use of VKAs, despite the advantages demonstrated for DOAC,^8,63^ the health benefits of AF screening are also reduced.

Although screening programmes can detect unknown AF, some criticism exists, and the limited evidence regarding the effects on health outcomes has been highlighted by the US Preventive Services Task Force.^64^ Indeed, up to now no randomized trial assessed the benefits and risks of anticoagulation among patients with screen-detected AF. However, benefits should be expected since the risk of stroke associated with screen-detected AF is comparable to that of clinically diagnosed AF^65-67^ and the composite endpoint of stroke incidence and death was reduced at 5 years in the geographic areas where AF screening was performed in the STROKESTOP trial.^68^ The STROKESTOP II study^69^ could not replicate this finding. However, the proportion of screen detected was lower in STROKESTOP II compared with STROKESTOP I, probably due to less intense monitoring for AF, as 40% of patients only had one registered single-lead ECG. A cost-conscious approach to new technologies and new preventive strategies has been advised across Europe^70^ and the implementation of interventions providing value, such as AF screening in appropriately selected patients, should be associated to appropriate reimbursement.^71^

Many AF patients suffer from multimorbidity and frailty, and therefor the need for multidisciplinary collaboration to achieve an integrated course of care, especially post-stroke, has been discussed in recent years.^72,73^

### Limitations

For several countries, the model parameter estimates are reliable; however, a potential limitation is the paucity of reliable data from certain countries. Specifically, there was a deficiency of country-specific unit cost data. This has, in some cases, meant that Swedish unit costs have been applied and adjusted to the countries in question by use of purchasing power parities. Due to differences in health care systems and other conditions, this might not correctly reflect the true costs in the specific countries. Furthermore, analyses are performed using a health economic simulation model, based on assumptions regarding certain parameters. The analyses assume that the stroke risk associated with screening-detected AF is similar to that of clinically detected AF used to derive the CHA_2_DS_2_-VASc. These assumptions may influence the results. However, the parameters are outlined in detail and can be substituted by more appropriate information once available. A notable strength of this study is its comprehensive approach, encompassing multiple European countries. The analyses apply to developed high-income western countries, so the results cannot be extrapolated to low- and middle-income countries.

### Policy implications

AF is a significant and costly public health issue in Europe, necessitating preventive measures such as screening. The principal implication of this pan-European cost-effectiveness study is the recommendation for the immediate implementation of AF screening programmes in Europe, particularly within healthcare systems analogous to those of the countries in this study. AF screening is associated with both health gains and long-term cost savings. The time to break even, defined as the point at which net cost equals zero, varies among the countries studied, ranging from 6 years in Ireland to 14 years in the Netherlands.

Future research should prioritize the comparison of various screening strategies to evaluate their respective health benefits, cost-effectiveness, and acceptance to patients, e.g. for routine heart rhythm assessment during healthcare contact as recommended in the current AF guidelines in all individuals aged ≥65.^9^ The preference for population-based, opportunistic, or targeted screening strategies may differ based on the distinct conditions prevalent in the countries under review. Additionally, it is imperative to investigate the optimal age for initial screening and to ascertain the suitable intervals for repeated screenings.

## Conclusions

This study indicates that population-based AF screening of 75-year-olds is a cost-effective strategy across eight European countries, meaning that adoption of such a strategy has the potential to make healthcare systems in these countries more efficient. The heterogeneity among European countries suggests that AF screening programmes may need to be tailored to the specific healthcare systems and conditions of each nation.

## Supplementary Material

qcaf099_Supplementary_Data

## Data Availability

Data available on request.
