# Supplementary material for: Cost-effectiveness of atrial fibrillation screening programmes across European nations
Source: Eur Heart J Qual Care Clin Outcomes. 2025 Aug 25;12(1):83–92. doi: 10.1093/ehjqcco/qcaf099 (PMC12770905; doi:10.1093/ehjqcco/qcaf099)
Supplement: qcaf099_Supplementary_Data [file qcaf099_supplementary_data.docx]

**Supplement to Cost-effectiveness of screening for atrial fibrillation across European countries.**

*Table S1. Distribution of initial drug treatment for new detected AF in the different countries.*

| **Country** | **DOAC** | **VKA** | **No OAC** | **References** |
| --- | --- | --- | --- | --- |
| **Denmark** | 85% | 10% | 5% | (1) |
| **Germany** | 74% | 19% | 7% | (2) |
| **Ireland** | 74% | 19% | 7% | (2)* |
| **Italy** | 80% | 15% | 5% | (3) |
| **The Netherlands** | 88.5% | 8.1% | 3.4% | (4) |
| **Serbia** | 60% | 30% | 10% | (5) |
| **Spain** | 60% | 35% | 5% | (6) |
| **Sweden** | 86.5% | 6.5% | 7% | (7, 8) |

**As no information was available for Ireland, the distribution in Germany was used also for Ireland.*

*Table S2. Treatment cessation rates in different countries. (9)*

| **Time period** | **Sweden** | **Denmark** | **Germany** | **Ireland** | **Italy** | **Nether**  **lands** | **Serbia** | **Spain** |
| --- | --- | --- | --- | --- | --- | --- | --- | --- |
| **0-3 months** | 5.3% | 5.6% | 8.4% | 5.2% | 5.8% | 8.4% | 5.8% | 5.0% |
| **4-6 months** | 2.6% | 3.2% | 6.3% | 3.4% | 4.1% | 6.0% | 4.1% | 2.4% |
| **7-9 months** | 1.3% | 1.6% | 2.7% | 1.8% | 1.8% | 2.6% | 1.8% | 1.3% |
| **10-12 months** | 1.9% | 1.3% | 3.0% | 2.2% | 2.0% | 3.0% | 2.0% | 1.8% |
| **Per cycle >1 year** | 0.2% | 0.1% | 0.3% | 0.7% | 0.2% | 0.2% | 0.2% | 0.1% |

*Table S3. Mortality after stroke. (10, 11)*

|  | **Ischaemic stroke** | | | | **Haemorrhagic stroke** | | | |
| --- | --- | --- | --- | --- | --- | --- | --- | --- |
| **Age (years)** | **0-3 Months** | **4-6 M** | **7-9 M** | **10-12 M** | **0-3 M** | **4-6 M** | **7-9 M** | **10-12 M** |
| **65** | 8.27% | 1.94% | 1.49% | 1.87% | 23.68% | 2.81% | 2.40% | 2.26% |
| **66** | 8.77% | 2.05% | 1.58% | 1.98% | 24.87% | 2.95% | 2.52% | 2.37% |
| **67** | 9.29% | 2.17% | 1.68% | 2.10% | 26.11% | 3.10% | 2.65% | 2.49% |
| **68** | 9.85% | 2.30% | 1.78% | 2.23% | 27.42% | 3.25% | 2.78% | 2.61% |
| **69** | 10.44% | 2.44% | 1.88% | 2.36% | 28.79% | 3.41% | 2.92% | 2.74% |
| **70** | 11.07% | 2.59% | 2.00% | 2.50% | 30.23% | 3.59% | 3.06% | 2.88% |
| **71** | 11.73% | 2.75% | 2.12% | 2.66% | 31.74% | 3.76% | 3.22% | 3.02% |
| **72** | 12.44% | 2.91% | 2.24% | 2.81% | 33.33% | 3.95% | 3.38% | 3.17% |
| **73** | 13.18% | 3.08% | 2.38% | 2.98% | 34.99% | 4.15% | 3.55% | 3.33% |
| **74** | 13.97% | 3.27% | 2.52% | 3.16% | 36.74% | 4.36% | 3.72% | 3.50% |
| **75** | 14.81% | 3.47% | 2.67% | 3.35% | 38.58% | 4.58% | 3.91% | 3.67% |
| **76** | 15.70% | 3.67% | 2.83% | 3.55% | 40.51% | 4.80% | 4.11% | 3.86% |
| **77** | 16.64% | 3.89% | 3.00% | 3.77% | 42.53% | 5.05% | 4.31% | 4.05% |
| **78** | 17.64% | 4.13% | 3.18% | 3.99% | 44.66% | 5.30% | 4.53% | 4.25% |
| **79** | 18.70% | 4.38% | 3.37% | 4.23% | 46.89% | 5.56% | 4.75% | 4.47% |
| **80** | 19.82% | 4.64% | 3.58% | 4.49% | 49.24% | 5.84% | 4.99% | 4.69% |
| **81** | 21.01% | 4.92% | 3.79% | 4.75% | 51.70% | 6.13% | 5.24% | 4.92% |
| **82** | 22.27% | 5.21% | 4.02% | 5.04% | 54.28% | 6.44% | 5.50% | 5.17% |
| **83** | 23.61% | 5.52% | 4.26% | 5.34% | 57.00% | 6.76% | 5.78% | 5.43% |
| **84** | 25.03% | 5.86% | 4.52% | 5.66% | 59.85% | 7.10% | 6.07% | 5.70% |
| **85+** | 26.53% | 6.21% | 4.79% | 6.00% | 59.85% | 7.10% | 6.07% | 5.70% |

*Table S4. Age- and sex specific standard mortality by country. (11)*

|  | **Sweden** | **Denmark** | **Germany** | **Ireland** | **Italy** | **Netherlands** | **Serbia** | **Spain** |
| --- | --- | --- | --- | --- | --- | --- | --- | --- |
| ***Men*** |  |  |  |  |  |  |  |  |
| **65** | 0.24% | 0.28% | 0.39% | 0.24% | 0.29% | 0.28% | 0.70% | 0.29% |
| **66** | 0.28% | 0.37% | 0.42% | 0.29% | 0.32% | 0.30% | 0.76% | 0.31% |
| **67** | 0.27% | 0.40% | 0.46% | 0.29% | 0.35% | 0.34% | 0.82% | 0.35% |
| **68** | 0.32% | 0.42% | 0.51% | 0.33% | 0.39% | 0.36% | 0.90% | 0.37% |
| **69** | 0.35% | 0.50% | 0.55% | 0.39% | 0.43% | 0.42% | 0.96% | 0.42% |
| **70** | 0.40% | 0.55% | 0.57% | 0.46% | 0.46% | 0.46% | 1.04% | 0.44% |
| **71** | 0.45% | 0.55% | 0.63% | 0.47% | 0.52% | 0.51% | 1.13% | 0.48% |
| **72** | 0.48% | 0.63% | 0.67% | 0.49% | 0.56% | 0.56% | 1.21% | 0.51% |
| **73** | 0.50% | 0.67% | 0.74% | 0.58% | 0.62% | 0.63% | 1.29% | 0.56% |
| **74** | 0.60% | 0.78% | 0.78% | 0.68% | 0.69% | 0.71% | 1.39% | 0.64% |
| **75** | 0.65% | 0.78% | 0.90% | 0.81% | 0.78% | 0.77% | 1.56% | 0.70% |
| **76** | 0.73% | 0.92% | 0.98% | 0.86% | 0.88% | 0.89% | 1.71% | 0.78% |
| **77** | 0.80% | 1.03% | 1.04% | 0.97% | 0.97% | 0.99% | 1.90% | 0.85% |
| **78** | 0.96% | 1.12% | 1.16% | 0.99% | 1.09% | 1.12% | 2.08% | 0.94% |
| **79** | 1.02% | 1.29% | 1.24% | 1.16% | 1.20% | 1.25% | 2.21% | 1.05% |
| **80** | 1.16% | 1.46% | 1.47% | 1.38% | 1.31% | 1.37% | 2.46% | 1.15% |
| **81** | 1.32% | 1.58% | 1.60% | 1.57% | 1.49% | 1.54% | 2.71% | 1.30% |
| **82** | 1.49% | 1.80% | 1.77% | 1.84% | 1.68% | 1.79% | 2.96% | 1.47% |
| **83** | 1.72% | 2.08% | 2.03% | 1.98% | 1.89% | 1.97% | 3.32% | 1.66% |
| **84** | 1.98% | 2.14% | 2.29% | 2.32% | 2.13% | 2.27% | 3.63% | 1.86% |
| **85** | 2.22% | 2.45% | 2.73% | 2.43% | 2.47% | 2.60% | 4.07% | 2.15% |
| **86** | 2.60% | 2.73% | 2.97% | 2.87% | 2.81% | 3.04% | 4.34% | 2.39% |
| **87** | 2.98% | 3.33% | 3.46% | 3.28% | 3.24% | 3.37% | 4.92% | 2.65% |
| **88** | 3.40% | 3.71% | 3.91% | 3.64% | 3.63% | 3.96% | 5.32% | 3.05% |
| **89** | 3.99% | 4.30% | 4.50% | 3.96% | 4.20% | 4.55% | 6.00% | 3.49% |
| **90** | 4.35% | 4.50% | 4.99% | 4.53% | 4.68% | 5.27% | 6.46% | 4.00% |
| **91** | 4.92% | 5.19% | 5.54% | 5.20% | 5.33% | 5.59% | 6.98% | 4.57% |
| **92** | 5.75% | 5.84% | 6.18% | 5.80% | 5.78% | 6.46% | 7.24% | 5.02% |
| **93** | 6.40% | 7.26% | 6.85% | 5.41% | 6.34% | 7.28% | 7.77% | 5.65% |
| **94** | 7.50% | 7.83% | 7.67% | 6.50% | 7.12% | 7.57% | 8.13% | 6.20% |
| **95** | 7.96% | 7.92% | 8.29% | 7.39% | 7.85% | 8.66% | 8.67% | 6.78% |
| **96** | 8.80% | 8.68% | 9.08% | 8.07% | 8.61% | 9.50% | 9.82% | 7.47% |
| **97** | 9.67% | 9.46% | 9.89% | 8.79% | 9.41% | 10.37% | 10.04% | 8.20% |
| **98** | 10.57% | 10.27% | 10.73% | 9.53% | 10.24% | 11.26% | 10.21% | 8.96% |
| **99** | 11.48% | 11.09% | 11.57% | 10.29% | 11.08% | 12.16% | 11.44% | 9.74% |
| **100** | 12.40% | 11.92% | 12.42% | 11.06% | 11.92% | 13.05% | 12.35% | 10.55% |
| **101** | 13.31% | 12.75% | 13.27% | 11.84% | 12.77% | 13.94% | 13.29% | 11.37% |
| **102** | 14.21% | 13.58% | 14.10% | 12.62% | 13.62% | 14.80% | 14.26% | 12.19% |
| **103** | 15.09% | 14.39% | 14.91% | 13.40% | 14.44% | 15.63% | 15.27% | 13.01% |
| **104** | 15.93% | 15.18% | 15.70% | 14.16% | 15.24% | 16.43% | 16.31% | 13.82% |
| **105** | 16.73% | 15.94% | 16.45% | 14.91% | 16.02% | 17.19% | 17.39% | 14.62% |
| **106** | 17.48% | 16.66% | 17.17% | 15.64% | 16.76% | 17.90% | 18.52% | 15.39% |
| **107** | 18.19% | 17.35% | 17.84% | 16.33% | 17.46% | 18.56% | 19.70% | 16.13% |
| **108** | 18.84% | 18.00% | 18.47% | 17.00% | 18.11% | 19.16% | 20.93% | 16.84% |
| **109** | 19.44% | 18.61% | 19.06% | 17.63% | 18.72% | 19.72% | 22.22% | 17.52% |
| **110** | 100.00% | 100.00% | 100.00% | 100.00% | 100.00% | 100.00% | 100.00% | 100.00% |
| ***Women*** |  |  |  |  |  |  |  |  |
| **65** | 0.16% | 0.20% | 0.20% | 0.15% | 0.15% | 0.20% | 0.37% | 0.13% |
| **66** | 0.16% | 0.25% | 0.22% | 0.19% | 0.17% | 0.22% | 0.42% | 0.14% |
| **67** | 0.21% | 0.25% | 0.23% | 0.21% | 0.20% | 0.25% | 0.43% | 0.16% |
| **68** | 0.22% | 0.29% | 0.26% | 0.23% | 0.21% | 0.27% | 0.49% | 0.17% |
| **69** | 0.24% | 0.29% | 0.29% | 0.26% | 0.23% | 0.30% | 0.56% | 0.19% |
| **70** | 0.25% | 0.35% | 0.32% | 0.28% | 0.25% | 0.32% | 0.61% | 0.20% |
| **71** | 0.28% | 0.36% | 0.35% | 0.34% | 0.28% | 0.36% | 0.65% | 0.21% |
| **72** | 0.33% | 0.38% | 0.38% | 0.36% | 0.32% | 0.39% | 0.76% | 0.23% |
| **73** | 0.34% | 0.42% | 0.42% | 0.41% | 0.36% | 0.42% | 0.86% | 0.27% |
| **74** | 0.41% | 0.44% | 0.45% | 0.44% | 0.39% | 0.48% | 0.95% | 0.31% |
| **75** | 0.47% | 0.55% | 0.51% | 0.51% | 0.45% | 0.55% | 1.06% | 0.32% |
| **76** | 0.50% | 0.61% | 0.58% | 0.64% | 0.50% | 0.60% | 1.20% | 0.38% |
| **77** | 0.61% | 0.69% | 0.61% | 0.64% | 0.58% | 0.68% | 1.42% | 0.43% |
| **78** | 0.64% | 0.74% | 0.71% | 0.75% | 0.64% | 0.75% | 1.59% | 0.50% |
| **79** | 0.75% | 0.84% | 0.77% | 0.84% | 0.73% | 0.87% | 1.82% | 0.57% |
| **80** | 0.80% | 0.94% | 0.91% | 0.94% | 0.82% | 0.98% | 2.03% | 0.63% |
| **81** | 0.92% | 1.09% | 1.02% | 1.01% | 0.95% | 1.07% | 2.20% | 0.75% |
| **82** | 1.09% | 1.25% | 1.17% | 1.14% | 1.10% | 1.30% | 2.52% | 0.85% |
| **83** | 1.25% | 1.32% | 1.39% | 1.52% | 1.28% | 1.42% | 2.93% | 1.01% |
| **84** | 1.43% | 1.53% | 1.62% | 1.50% | 1.48% | 1.61% | 3.20% | 1.20% |
| **85** | 1.63% | 1.80% | 1.94% | 1.99% | 1.74% | 1.94% | 3.62% | 1.41% |
| **86** | 1.85% | 2.08% | 2.18% | 2.27% | 1.99% | 2.24% | 4.00% | 1.65% |
| **87** | 2.19% | 2.37% | 2.58% | 2.48% | 2.32% | 2.64% | 4.56% | 1.84% |
| **88** | 2.42% | 2.83% | 2.96% | 2.92% | 2.68% | 3.13% | 4.93% | 2.18% |
| **89** | 2.80% | 3.13% | 3.40% | 3.21% | 3.07% | 3.51% | 5.51% | 2.52% |
| **90** | 3.29% | 3.71% | 3.95% | 3.35% | 3.56% | 4.13% | 6.07% | 2.95% |
| **91** | 3.78% | 4.33% | 4.49% | 3.97% | 4.06% | 4.77% | 6.51% | 3.36% |
| **92** | 4.42% | 4.67% | 5.09% | 4.15% | 4.47% | 5.16% | 6.91% | 3.80% |
| **93** | 4.82% | 5.55% | 5.66% | 4.90% | 5.14% | 6.05% | 7.52% | 4.27% |
| **94** | 5.67% | 5.89% | 6.35% | 5.82% | 5.60% | 6.55% | 8.08% | 4.82% |
| **95** | 6.19% | 6.70% | 7.09% | 6.37% | 6.33% | 7.44% | 8.49% | 5.42% |
| **96** | 6.92% | 7.45% | 7.90% | 7.07% | 7.06% | 8.29% | 9.18% | 6.10% |
| **97** | 7.70% | 8.26% | 8.75% | 7.82% | 7.84% | 9.17% | 9.55% | 6.82% |
| **98** | 8.52% | 9.10% | 9.64% | 8.60% | 8.66% | 10.09% | 10.61% | 7.60% |
| **99** | 9.38% | 9.97% | 10.56% | 9.41% | 9.51% | 11.03% | 9.49% | 8.42% |
| **100** | 10.27% | 10.86% | 11.49% | 10.25% | 10.39% | 11.98% | 10.44% | 9.28% |
| **101** | 11.18% | 11.77% | 12.43% | 11.10% | 11.29% | 12.94% | 11.42% | 10.17% |
| **102** | 12.10% | 12.68% | 13.36% | 11.96% | 12.20% | 13.88% | 12.44% | 11.08% |
| **103** | 13.01% | 13.58% | 14.28% | 12.83% | 13.10% | 14.80% | 13.49% | 12.00% |
| **104** | 13.91% | 14.46% | 15.17% | 13.68% | 13.99% | 15.68% | 14.59% | 12.91% |
| **105** | 14.80% | 15.32% | 16.03% | 14.52% | 14.86% | 16.53% | 15.73% | 13.82% |
| **106** | 15.65% | 16.14% | 16.84% | 15.34% | 15.70% | 17.33% | 16.91% | 14.71% |
| **107** | 16.46% | 16.92% | 17.61% | 16.12% | 16.50% | 18.07% | 18.15% | 15.57% |
| **108** | 17.24% | 17.66% | 18.32% | 16.86% | 17.26% | 18.76% | 19.45% | 16.39% |
| **109** | 17.96% | 18.34% | 18.97% | 17.57% | 17.97% | 19.38% | 20.81% | 17.17% |
| **110** | 100.00% | 100.00% | 100.00% | 100.00% | 100.00% | 100.00% | 100.00% | 100.00% |

The latest available tables are used for each country. Sweden (2023), Denmark (2023), Germany (2020), Ireland (2022), Italy (2021), Netherlands (2022), Serbia (2022) and Spain (2023).

*Table S5. Country-specific costs (€)*

| **Cost item** | **Sweden** | **Denmark** | **Germany** | **Ireland** | **Italy** | **NL** | **Serbia^[[1]](#footnote-2)^** | **Spain** |
| --- | --- | --- | --- | --- | --- | --- | --- | --- |
| **Screening** |  |  |  |  |  |  |  |  |
| **Invitation** | 2 (12) | 1.57 (12) | 2.5 (13) | 1 (12) | 1.3 (12) | 1.75 (14) | 0.4 | 1 (12) |
| **Device cost** | 235 (15) | 36 (1) | 28 (2) | 27 (15) | 220 (3) | 233 (14) | 42 | 136 (16) |
|  |  |  |  |  |  |  |  |  |
| **Newly detected AF** |  |  | (2) |  |  |  |  |  |
| **No. visits cardiologist** | 1 (17) | 0.5 (1) |  |  | 1 (17) |  | 1 | 1 (17) |
| **No. visits to primary health care (PHC)** | 1 (17) | 3 (1) | 1 |  | 1 (17) |  | 1 | 1 (17) |
| **Unit cost card. visit** | 265 (18) | 112 (19) |  |  | 125 (20) |  | 50 | 150 (21) |
| **Unit cost PHC visit** | 193 (22) | 21 (19) | 13 |  | 26 (23) |  | 35 | 40 (21) |
| ***Total cost*** | *470* | *328* | *72* | *225 (24)* | *151* | *388 (14)* | *85* | *190* |
|  |  |  |  |  |  |  |  |  |
| **DOAC** |  |  |  |  |  |  |  |  |
| **Medication (per cycle)** | 158 (25) | 220 (26) | 260 (27) | 198 (28) | 127 (3) | 196 (29) | 127 | 265 (30) |
| **No. visits cardiologist*** | 0.5 (17) | 0 (1) | 0 (2) |  | 0.5 (3) | 1 (31) | 0.5 | 0.5 (17) |
| **No. visits PHC*** | 1.25 (17) | 1 (1) |  |  | 1.25 (3) | 0.5 (31) | 1.25 | 1.25 (17) |
| **Unit cost card. visit** | 186 (32) | 265 (19) |  |  | 125 (20) | 107 (31) | 34 | 150 (21) |
| **Unit cost PHC visit** | 193 (22) | 21 (19) |  |  | 26 (23) | 39 (31) | 35 | 40 (21) |
| **Cost physician visits (per cycle)** | 83 | 5 | 13 (33) | 58^[[2]](#footnote-3)^ | 24 | 32 | 15 | 31 |
| ***Cost DOAC (per cycle)*** | *241* | *225* | *273* | *256 (34)* | *151* | *228* | *142* | *296* |
|  |  |  |  |  |  |  |  |  |
| **VKA (Warfarin)** |  |  |  |  |  |  |  |  |
| **Medication (per cycle)** | 16 (25) | 32 (26) | 39 (27) | 7 (28) | 5 (35) | 14 (3) | 5 | 5 (30) |
| **No. visits cardiologist*** | 0.5 (17) | 0 (1) |  |  | 1 (3) | 1 (31) | 0.5 |  |
| **No. visits PHC*** | 0.5 (17) | 0 (1) |  |  | 1 (3) | 0.5 (31) | 0.5 |  |
| **Unit cost card. visit** | 186 (32) | 265 (19) |  |  | 125 (20) | 107 (31) | 34 |  |
| **Unit cost PHC visit** | 193 (22) | 21 (19) |  |  | 26 (23) | 39 (31) | 35 |  |
| **Cost INR (per cycle)** | 87 | 279 (1) |  | 111^[[3]](#footnote-4)^ | 108 (35) | 42 (31) | 16 | 120 (36) |
| ***Cost Warfarin (per cycle)*** | *150* | *311* | *82 (13)* | *118 (34)* | *151* | *87* | *30* | *125* |
|  |  |  |  |  |  |  |  |  |
| **Post-IS*** costs (per cycle)** | (37) | (38) | (13, 39) | (34) | (40, 41) | (42) |  | (43, 44) |
| **Inpatient year 1** | 5215 | 3904 | 1505 | 2451 | 3431 | 12780 ^x x^ | 943 | 1641 |
| **Outpatient year 1** | 1006 | 122 | 3087^ | 664 |  |  | 182 | 5819 |
| **Other^[[4]](#footnote-5)^ care year 1** |  |  |  |  | 2355 |  |  |  |
| **Home care, special housing year 1** | 4764 | 891 | 512 | 979 |  |  | 861 |  |
| **Out-/inpat. >year 1** | 1758 | 318 | 1320^ | 1146 | 2655 | 3162 ^x x^ | 318 | 588 |
| **Direct non-healthcare costs >year 1** |  |  |  |  | 7845 |  |  | 3087 |
| **Home care, special housing >year 1** | 8678 | 1574 | 353 | 3841 |  |  | 1569 |  |
| **Post-HS*** costs (per cycle)** |  |  |  |  |  |  |  |  |
| **Inpatient year 1** | 8127 | 4519 | 3296 | 2402 | 5521^x^ | 14073 ^x x^ | 1469 | 1365 |
| **Outpatient year 1** | 894 | 37 | 3087^ | 891 |  |  | 162 | 6513 |
| **Informal care year 1** |  |  |  |  | 2114 |  |  |  |
| **Paid care year 1** |  |  |  |  | 241 |  |  |  |
| **Home care, special housing year 1** | 4634 | 855 | 512 | 953 |  |  | 838 |  |
| **Out-/inpat. >year 1** | 1664 | 322 | 1320^ | 1232 | 2655 | 3756 ^x x^ | 301 | 588 |
| **Direct non-healthcare costs >year 1** |  |  |  |  | 7845 |  |  |  |
| **Home care, special housing >year 1** | 9902 | 1841 | 353 | 4342 |  |  | 1790 | 3087 |
| **Health events** | (45) | (1, 45, 46) | (45) | (34) | (45) | (14, 45) |  | (45) |
| **Systemic embolism** | 4338 | 3696 | 2640 | 3975 | 3123 | 1992 | 784 | 2723 |
| **Other intracranial bleeding** | 5661 | 4597 | 3446 | 2669 | 4075 | 4614 | 1024 | 3553 |
| **Extracranial bleeding** | 4775 | 3877 | 2906 | 2669 | 3437 | 10823 | 863 | 2997 |

*Per year.

**INR=International Normalized Ratio.

***IS=ischemic stroke, HS=haemorrhagic stroke.

^Including rehabilitation.

^x^ Outpatient- and inpatient care.

^xx^ Including outpatient- and inpatient care and home care/special housing. In calculations, HS is 10% more costly the first year and 19% more costly after the year compared to IS, based on a publication from the Netherlands on highly functional Stroke-patients (47).

*Table S6. Country-specific utility weights by age in the AFFECT-EU model.*

| **Age** | **Sweden (48)** | **Denmark (49)** | **Germany (49)** | **Italy (49)** | **Ireland (UK) (49)** | **Netherlands (49)** | **Serbia (Italy) (49)** | **Spain (49)** |
| --- | --- | --- | --- | --- | --- | --- | --- | --- |
| **65** | 0.903 | 0.859 | 0.907 | 0.920 | 0.789 | 0.888 | 0.920 | 0.896 |
| **66** | 0.899 | 0.856 | 0.903 | 0.917 | 0.787 | 0.888 | 0.917 | 0.895 |
| **67** | 0.895 | 0.854 | 0.900 | 0.914 | 0.785 | 0.887 | 0.914 | 0.894 |
| **68** | 0.891 | 0.852 | 0.897 | 0.910 | 0.783 | 0.887 | 0.910 | 0.893 |
| **69** | 0.887 | 0.849 | 0.894 | 0.907 | 0.781 | 0.886 | 0.907 | 0.892 |
| **70** | 0.883 | 0.847 | 0.891 | 0.904 | 0.779 | 0.886 | 0.904 | 0.891 |
| **71** | 0.879 | 0.842 | 0.886 | 0.898 | 0.774 | 0.880 | 0.898 | 0.880 |
| **72** | 0.875 | 0.836 | 0.881 | 0.891 | 0.768 | 0.875 | 0.891 | 0.869 |
| **73** | 0.871 | 0.831 | 0.875 | 0.885 | 0.763 | 0.869 | 0.885 | 0.858 |
| **74** | 0.867 | 0.826 | 0.870 | 0.878 | 0.758 | 0.864 | 0.878 | 0.847 |
| **75** | 0.863 | 0.821 | 0.865 | 0.872 | 0.753 | 0.858 | 0.872 | 0.836 |
| **76** | 0.847 | 0.815 | 0.860 | 0.865 | 0.747 | 0.852 | 0.865 | 0.825 |
| **77** | 0.830 | 0.810 | 0.855 | 0.859 | 0.742 | 0.847 | 0.859 | 0.814 |
| **78** | 0.814 | 0.805 | 0.849 | 0.852 | 0.737 | 0.841 | 0.852 | 0.803 |
| **79** | 0.797 | 0.799 | 0.844 | 0.846 | 0.731 | 0.836 | 0.846 | 0.792 |
| **80** | 0.781 | 0.794 | 0.839 | 0.839 | 0.726 | 0.830 | 0.839 | 0.781 |
| **81** | 0.765 | 0.789 | 0.834 | 0.833 | 0.721 | 0.824 | 0.833 | 0.770 |
| **82** | 0.748 | 0.783 | 0.829 | 0.826 | 0.715 | 0.819 | 0.826 | 0.759 |
| **83** | 0.732 | 0.778 | 0.823 | 0.820 | 0.710 | 0.813 | 0.820 | 0.748 |
| **84** | 0.715 | 0.773 | 0.818 | 0.813 | 0.705 | 0.808 | 0.813 | 0.737 |
| **85+** | 0.699 | 0.768 | 0.813 | 0.807 | 0.700 | 0.802 | 0.807 | 0.726 |

*Table S7. Discount rates applied in analyses of different countries.*

| **Discount rate** | **Sweden** | **Denmark** | **Germany** | **Ireland** | **Italy** | **NL** | **Serbia** | **Spain** |
| --- | --- | --- | --- | --- | --- | --- | --- | --- |
| **Costs** | 3% | 1.5%* | 5% | 5% | 3% | 4% | 3% | 3% |
| **Utilities (QALYs)** | 3% | 1.5%* | 5% | 5% | 3% | 1.5% | 3% | 3% |

*1.5% for people >70 years, 2.5% for people ≤70 years

*Table S8. Sensitivity analysis of country-specific results with 25-50% reduced stroke risk for undetected and screen-detected AFs compared to clinically diagnosed AFs, per 1000 persons invited to AF screening compared to no screening.*

|  | **Reduced stroke risk** | **Strokes avoided** | **Life-years gained** | **QALYs gained** | **Additional costs (€)** | **ICER (€/QALY)** |
| --- | --- | --- | --- | --- | --- | --- |
| Denmark | 0% (base case) | 3.4 | 3.3 | 5.5 | -56 065 | Dominant |
|  | 25% | 2.5 | 2.3 | 4.0 | -18 698 | Dominant |
|  | 50% | 1.5 | 1.4 | 2.5 | 19 152 | 7 765 |
| Germany | 0% (base case) | 2.8 | 2.4 | 4.1 | -29 667 | Dominant |
|  | 25% | 1.9 | 1.7 | 2.9 | -2 427 | Dominant |
|  | 50% | 1.1 | 0.9 | 1.7 | 25 164 | 15 075 |
| Ireland | 0% (base case) | 2.8 | 2.5 | 4.1 | -167 934 | Dominant |
|  | 25% | 2.0 | 1.8 | 2.9 | -102 702 | Dominant |
|  | 50% | 1.1 | 1.0 | 1.7 | -36 696 | Dominant |
| Italy | 0% (base case) | 3.3 | 3.5 | 5.9 | -494 753 | Dominant |
|  | 25% | 2.4 | 2.5 | 4.3 | -316 181 | Dominant |
|  | 50% | 1.4 | 1.5 | 2.6 | -135 175 | Dominant |
| The Netherlands | 0% (base case) | 2.8 | 3.3 | 5.7 | -37 326 | Dominant |
|  | 25% | 2.0 | 2.3 | 4.0 | 22 789 | 5 644 |
|  | 50% | 1.1 | 1.3 | 2.4 | 83 729 | 35 627 |
| Serbia | 0% (base case) | 2.3 | 1.6 | 3.1 | -30 666 | Dominant |
|  | 25% | 1.1 | 0.7 | 1.5 | 5 832 | 3 860 |
|  | 50% | -0.1 | -0.1 | -0.1 | 42 469 | Inferior |
| Spain | 0% (base case) | 3.4 | 3.7 | 6.1 | -147 084 | Dominant |
|  | 25% | 2.4 | 2.7 | 4.4 | -70 958 | Dominant |
|  | 50% | 1.5 | 1.6 | 2.7 | 6 278 | 2 333 |
| Sweden | 0% (base case) | 3.5 | 3.8 | 6.0 | -531 474 | Dominant |
|  | 25% | 2.5 | 2.7 | 4.4 | -337 097 | Dominant |
|  | 50% | 1.6 | 1.7 | 2.7 | -140 176 | Dominant |


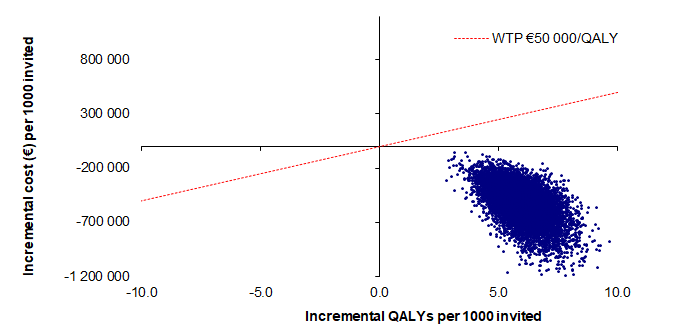


*Figure S1. Results from probabilistic cost-effectiveness analysis for Sweden.*


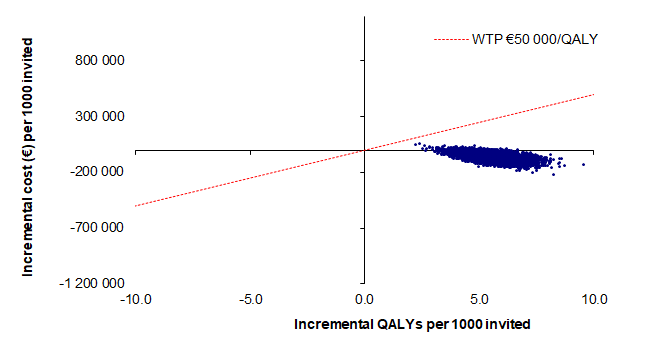


*Figure S2. Results from probabilistic cost-effectiveness analysis for Denmark.*


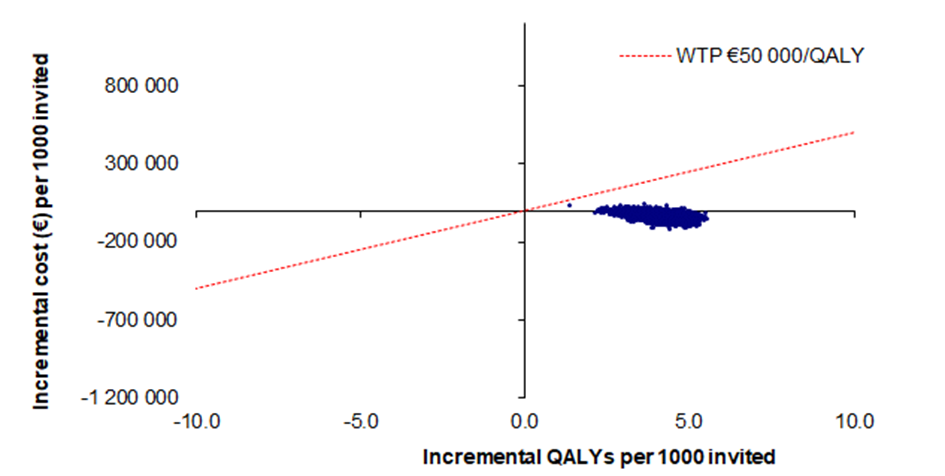


*Figure S3. Results from probabilistic cost-effectiveness analysis for Germany.*


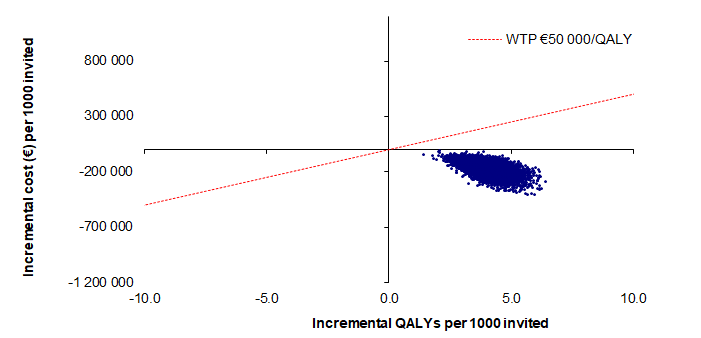


*Figure S4. Results from probabilistic cost-effectiveness analysis for Ireland*


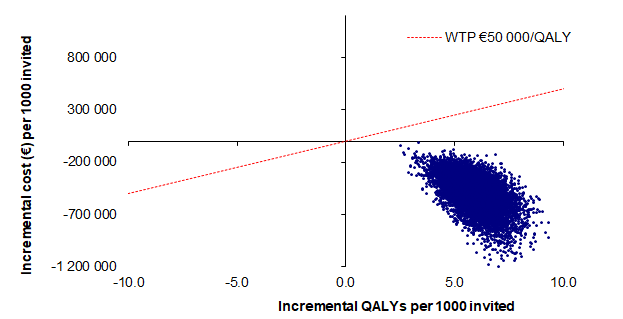


*Figure S5. Results from probabilistic cost-effectiveness analysis for Italy.*


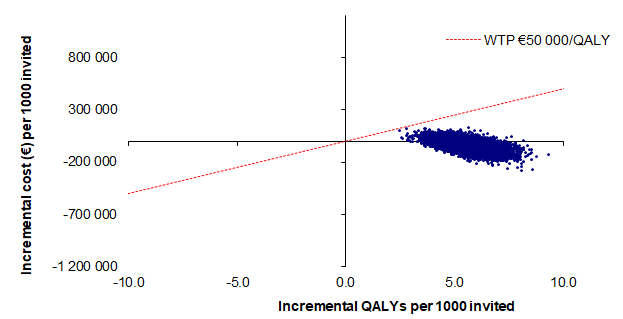


*Figure S6. Results from probabilistic cost-effectiveness analysis for the Netherlands.*


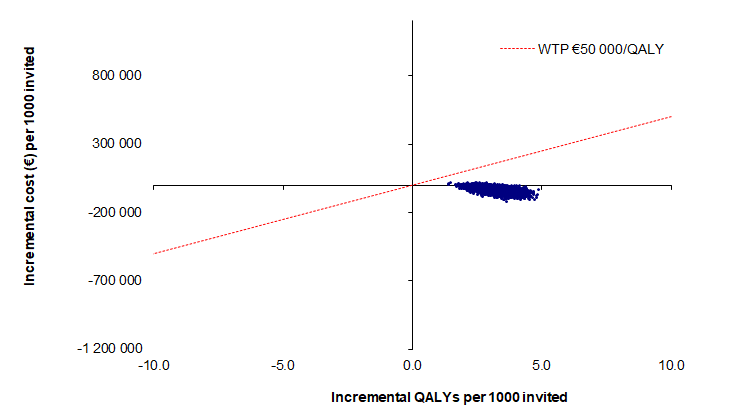


*Figure S7. Results from probabilistic cost-effectiveness analysis for Serbia.*


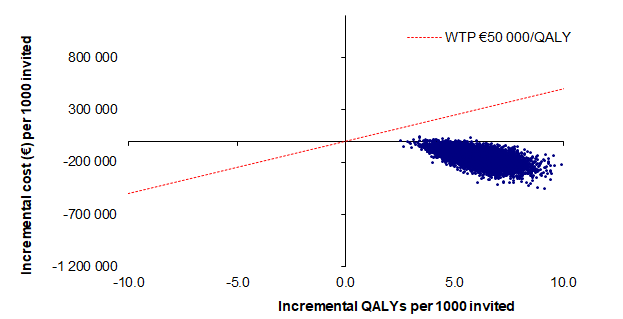


*Figure S8. Results from probabilistic cost-effectiveness analysis for Spain.*

**References for Supplement**

1. Svendsen JH. personal comunication. 30 march. 2022.

2. Schnabel R. personal communication. 22 february. 2022.

3. Boriani G. personal communication. 12 march. 2022.

4. Seelig J, Trinks-Roerdink E, Chu G, Pisters R, Theunissen L, Trines S, et al. Determinants of label non-adherence to non-vitamin K oral anticoagulants in patients with newly diagnosed atrial fibrillation. European Heart Journal Open. 2022;2(3).

5. Potpara T. Personal communication Feb 25. 2025.

6. Cayuelas JMA, Checa JR, Rodríguez FMS, Tolosa JN, Balsalobre MCG, Carretero ZS, et al. Aumento de la prescripcion de anticoagulantes orales directos y correlacion con la disminucion de ingresos hospitalarios per hemorragia intracraneal en pacientes con fibrilacion auricular durante la pandemia de COVID-19. Revista Española de Cardiología (English Edition). 2023;76:2-1098.

7. Svennberg E, Engdahl J, Al-Khalili F, Friberg L, Frykman V, Rosenqvist M. Mass Screening for Untreated Atrial Fibrillation. Circulation. 2015;131(25):2176-84.

8. Hjemdahl P, Braunschweig F, Holmström M, Johnsson H, von Euler M, Wallén H, et al. [Improved stroke prevention in atrial fibrillation: the Stockholm experience of the introduction of NOACs]. Lakartidningen. 2018;115.

9. Komen JJ, Heerdink ER, Klungel OH, Mantel-Teeuwisse AK, Forslund T, Wettermark B, et al. Long-term persistence and adherence with non-vitamin K oral anticoagulants in patients with atrial fibrillation and their associations with stroke risk. European Heart Journal - Cardiovascular Pharmacotherapy. 2020.

10. Sennfält S, Norrving B, Petersson J, Ullberg T. Long-Term Survival and Function After Stroke. Stroke. 2019;50(1):53-61.

11. Human Mortality Database. University of California BU, and Max Planck Institute for Demographic Research (Germany). Available at [www.mortality.org](https://liuonline-my.sharepoint.com/personal/johly06_liu_se/Documents/Projekt/Affect_EU/CE-report/För%20inskick/www.mortality.org) or [www.humanmortality.de](https://liuonline-my.sharepoint.com/personal/johly06_liu_se/Documents/Projekt/Affect_EU/CE-report/För%20inskick/www.humanmortality.de) (data downloaded on 26 oct 2021). [

12. Levin LA, Husberg M, Sobocinski PD, Kull VF, Friberg L, Rosenqvist M, et al. A cost-effectiveness analysis of screening for silent atrial fibrillation after ischaemic stroke. Europace. 2015;17(2):207-14.

13. Malavasi VL, Zoccali C, Brandi MC, Micali G, Vitolo M, Imberti JF, et al. Cognitive impairment in patients with atrial fibrillation: Implications for outcome in a cohort study. Int J Cardiol. 2021;323:83-9.

14. Jacobs MS, Kaasenbrood F, Postma MJ, van Hulst M, Tieleman RG. Cost-effectiveness of screening for atrial fibrillation in primary care with a handheld, single-lead electrocardiogram device in the Netherlands. Europace. 2018;20(1):12-8.

15. Lyth J, Svennberg E, Bernfort L, Aronsson M, Frykman V, Al-Khalili F, et al. Cost-effectiveness of population screening for atrial fibrillation: the STROKESTOP study. European Heart Journal. 2023;44(3):196-204.

16. Welton NJ, McAleenan A, Thom HH, Davies P, Hollingworth W, Higgins JP, et al. Screening strategies for atrial fibrillation: a systematic review and cost-effectiveness analysis. Health Technol Assess. 2017;21(29):1-236.

17. Friberg L. Personal communication. 2021.

18. Socialstyrelsen. [National Board of Health and Welfare] NORD-DRG E830.

19. Sundheddatastyrelsen. In: [Sundheddatastyrelsen] TDhda, editor.

20. SanitáPrivata. Quanto costauna visita cardiologica? 2020.

21. Expatica. Going to the doctor in Spain: a guide for expats. 2022.

22. Lin H, Wang M, Brody JA, Bis JC, Dupuis J, Lumley T, et al. Strategies to design and analyze targeted sequencing data: cohorts for Heart and Aging Research in Genomic Epidemiology (CHARGE) Consortium Targeted Sequencing Study. Circulation Cardiovascular genetics. 2014;7(3):335-43.

23. Omceoteramo. Tariffario minimo nazionale in euro. 2022.

24. Healy P. NCO-01-2022 Phase 2 of the structured chronic disease management programme in 2022. 2022.

25. FASS (Pharmaceutical Specialties in Sweden). Pharmaceutical prices in Sweden. <http://www.fass.se> (9 Sept 2021 dla.

26. Laegemiddelstyrelsen. [Danish medicines agency] Medicinpriser [Available from: <https://www.medicinpriser.dk/default.aspx>.

27. Schnabel RB, Post F, Blankenberg S. [Diagnosis of acute coronary syndrome]. Deutsche medizinische Wochenschrift (1946). 2014;139 Suppl 1:S9-12.

28. HSE. PCRS Search reimbursable items. 2021.

29. Dutch healthcare institute. Medicijnkosten. 2022.

30. Oyagüez I, Suárez C, López-Sendón JL, González-Juanatey JR, De Andrés-Nogales F, Suárez J, et al. Cost-Effectiveness Analysis of Apixaban Versus Edoxaban in Patients with Atrial Fibrillation for Stroke Prevention. PharmacoEconomics - Open. 2020;4(3):485-97.

31. Jacobs M. personal communication. 5 May. 2022.

32. Socialstyrelsen. [National Board of Health and Welfare] NORD-DRG E800.

33. Schnabel RB. Common genetic variation of blood pressure traits and their relation to end-organ damage. Circulation Cardiovascular genetics. 2011;4(6):712-3.

34. Moran PS, Teljeur C, Harrington P, Smith SM, Smyth B, Harbison J, et al. Cost-Effectiveness of a National Opportunistic Screening Program for Atrial Fibrillation in Ireland. Value Health. 2016;19(8):985-95.

35. Mennini F, Russo S, Marcellusi A. Budget impact analysis resulting from the use of dabigatran etexilate in preventing stroke in patients with non-valvular atrial fibrillation in Italy. Farmeconomia Health economics and therapeutic pathways. 2012;13:121.

36. González-Juanatey JR, Álvarez-Sabin J, Lobos JM, Martínez-Rubio A, Reverter JC, Oyagüez I, et al. Cost-effectiveness of Dabigatran for Stroke Prevention in Non-valvular Atrial Fibrillation in Spain. Revista Española de Cardiología (English Edition). 2012;65(10):901-10.

37. Lekander I, Willers C, Von Euler M, Lilja M, Sunnerhagen KS, Pessah-Rasmussen H, et al. Relationship between functional disability and costs one and two years post stroke. PLOS ONE. 2017;12(4):e0174861.

38. Jakobsen M, Kolodziejczyk C, Fredslund EK, Poulsen PB, Dybro L, Johnsen SP. Societal Costs of First-Incident Ischemic Stroke in Patients with Atrial Fibrillation—A Danish Nationwide Registry Study. Value in Health. 2016;19(4):413-8.

39. Kolominsky-Rabas PL, Heuschmann PU, Marschall D, Emmert M, Baltzer N, NeundöRfer B, et al. Lifetime Cost of Ischemic Stroke in Germany: Results and National Projections From a Population-Based Stroke Registry. Stroke. 2006;37(5):1179-83.

40. Fattore G, Torbica A, Susi A, Giovanni A, Benelli G, Gozzo M, et al. The social and economic burden of stroke survivors in Italy: a prospective, incidence-based, multi-centre cost of illness study. BMC Neurol. 2012;12:137.

41. Chiumente M, Gianino MM, Minniti D, Mattei TJ, Spass B, Kamal KM, et al. Burden of Stroke in Italy: An Economic Model Highlights Savings Arising from Reduced Disability following Thrombolysis. International Journal of Stroke. 2015;10(6):849-55.

42. Baeten SA, van Exel NJ, Dirks M, Koopmanschap MA, Dippel DW, Niessen LW. Lifetime health effects and medical costs of integrated stroke services - a non-randomized controlled cluster-trial based life table approach. Cost Eff Resour Alloc. 2010;8:21.

43. Lopez-Bastida J, Oliva Moreno J, Worbes Cerezo M, Perestelo Perez L, Serrano-Aguilar P, Montón-Álvarez F. Social and economic costs and health-related quality of life in stroke survivors in the Canary Islands, Spain. BMC Health Services Research. 2012;12(1):315.

44. Alvarez-Sabín J, Quintana M, Masjuan J, Oliva-Moreno J, Mar J, Gonzalez-Rojas N, et al. Economic impact of patients admitted to stroke units in Spain. The European Journal of Health Economics. 2017;18(4):449-58.

45. Lanitis T, Kongnakorn T, Jacobson L, De Geer A. Cost-effectiveness of Apixaban versus Warfarin and Aspirin in Sweden for Stroke Prevention in Patients with Atrial Fibrillation. Thrombosis Research. 2014;134(2):278-87.

46. Langkilde LK, Bergholdt Asmussen M, Overgaard M. Cost-effectiveness of dabigatran etexilate for stroke prevention in non-valvular atrial fibrillation. Applying RE-LY to clinical practice in Denmark. Journal of Medical Economics. 2012;15(4):695-703.

47. van Mastrigt G, van Heugten C, Visser-Meily A, Bremmers L, Evers S. Estimating the Burden of Stroke: Two-Year Societal Costs and Generic Health-Related Quality of Life of the Restore4Stroke Cohort. Int J Environ Res Public Health. 2022;19(17).

48. Burstrom K, Johannesson M, Diderichsen F. A comparison of individual and social time trade-off values for health states in the general population. Health Policy. 2006;76(3):359-70.

49. Janssen MF, Szende A, Cabases J, Ramos-Goñi JM, Vilagut G, König HH. Population norms for the EQ-5D-3L: a cross-country analysis of population surveys for 20 countries. The European Journal of Health Economics. 2019;20(2):205-16.

1. Serbian costs have been consistently calculated based on Swedish data. [↑](#footnote-ref-2)
2. Derived from the total cost of 256 and cost for DOAC medication, i.e., 256 minus 198. [↑](#footnote-ref-3)
3. Derived from the total cost of 118 and cost for VKA medication, i.e., 118 minus 7. [↑](#footnote-ref-4)
4. Other care consists of informal care, paid care and homecare. [↑](#footnote-ref-5)
